# The Efficacy of Rosehip Oil Emulsion as a Pro-Healing Herbal Medicine for the Treatment of Induced Mucosal Ulcer (Cell Culture and Experimental Study)

**DOI:** 10.3290/j.ohpd.c_2158

**Published:** 2025-08-05

**Authors:** Ola Abdel Moneim Dewedar, Doaa Adel Habba, Heba Abdelfatah Zaki, Enas Ahmed Elamin, Amal Ali Ibrahim, Omneya Emam Ahmed

**Affiliations:** a Ola Abdel Moneim Dewedar Lecturer of Oral and Dental Biology, Faculty of Dental Medicine for Girls, Al Azhar University, Cairo, Egypt. Conceived and designed the idea, conducted the practical part of the experiment, revised and edited the final version of the manuscript.; b Doaa Adel Habba Assistant Professor of Oral Pathology, Department of Oral and Maxillofacial Surgery and Diagnostic Sciences, Faculty of Dentistry, Najran University, Najran, Kingdom of Saudi Arabia; Lecturer of Oral and Dental Pathology, Faculty of Dental Medicine for Girls, Al Azhar University, Cairo, Egypt. Conducted the practical part of the experiment, prepared the figures, interpreted the results and drafted the main manuscript, revised and edited the final version of the manuscript.; c Heba Abdelfatah Zaki Lecturer of Oral and Dental Biology. Faculty of Dental Medicine for Girls, Al Azhar University, Cairo, Egypt; Lecturer of Oral and Dental Biology, Faculty of Oral & Dental Medicine, Nile Valley University, Faiyum, Egypt. Conducted the practical part of the experiment; d Enas Ahmed Elamin Assistant Professor of Dental Public Health, Department of Preventive Dentistry, Faculty of Dentistry, Najran University, Najran, Kingdom of Saudi Arabia. Assistant Professor of Dental Public Health, Shendi University, Sudan. Participated in data and statistical analysis.; e Amal Ali Ibrahim Lecturer of Oral Medicine, Periodontology, Oral Diagnosis and Dental Radiology, Faculty of Dental Medicine for Girls, Alazhar University, Cairo, Egypt. Participated in data and statistical analysis, prepared the figures, interpreted the results and drafted the main manuscript.; f Omneya Emam Ahmed Lecturer of Oral Medicine, Periodontology, 0ral Diagnosis and Radiology Department, Faculty of Dental Medicine for Girls, Al Azhar University, Cairo, Egypt. Conceived and designed the idea, participated in data and statistical analysis, prepared the figures, interpreted the results and drafted the main manuscript.

**Keywords:** rosehip oil, anti-inflammatory activity, oral ulcer, wound healing.

## Abstract

**Purpose:**

This study aimed to determine the impact of rose hip oil emulsion (ROE) on the healing of oral ulcers. The study first utilised the MTT kit to examine the effect of 20 mg/mL ROE on human gingival fibroblast (HGF) proliferation at the cellular level and its effect in treating oral mucosal ulcers at the experimental level.

**Materials and Methods:**

Sixty-six adult male rats with a chemically induced ulcer in the buccal mucosa. The animals were distributed randomly into two groups: a control group that had not received any treatment and a test group that was treated with topical ROE 3 times per day for 10 days. The samples were obtained on day 3, day 7, and day 10, and then the tissue staining was done using hematoxylin and eosin (H&E) and histomorphometric analysis.

**Results:**

The findings of this study demonstrated from cellular investigations that 20 mg/mL ROE can efficiently stimulate HGF proliferation at 24, 48, and 72 h. The animal study results revealed that ROE could substantially boost the healing of the induced ulcer model by lowering the inflammatory cells and extensively promoting collagen formation within the ulcer site on days 3 and 7.

**Conclusion:**

The topical application of 20 mg/mL ROE possesses anti-inflammatory properties, increasing the epithelium thickness and promoting collagen production and remodelling. Therefore, the rosehip oil emulsion can be considered an effective pro-healing agent that accelerates the healing of oral ulcers.

Oral mucosal ulcers are the most prevalent oral lesions, often produced by an epithelial defect, and appear as round sores with a yellow-white appearance.^8,34^ Local trauma, either physical, chemical, or thermal trauma, is one of the most frequent causes of mouth ulcers^7^. It is challenging for dentists to diagnose oral mucosa lesions because many ulcerated lesions share the same clinical and histological characteristics.^40^

There are two types of interventions available for oral ulcers. Physical interventions such as sprays and patches^43,35^ and medication therapy involve the application of anti-inflammatory agents and pharmaceuticals that aid in tissue healing. However, these drugs do not significantly shorten the time ulcers take to heal.^17,20^ Furthermore, very few anti-oral mucosal ulcer medications are available at pharmacies, including synthetic drugs and herbal alternatives.^27^

Additionally, the medical compliance of patients is often minimal because of the limited effectiveness of drugs and their continual application. Hence, developing a medication that may effectively decrease pain, remain on the ulcer site for an extended period, and help heal oral ulcers by improving tissue repair and lowering inflammation is required.^10^

The rosehip (*Rosa canina L*.) is a Rosaceae family member and an essential supplier of multiple nutrients.^38^ Rosehip oil (RO) is a significant source of vitamins such as A, B_1_, B_2_, and K and minerals (Ca, Fe, K, Mg, and Na).^26^ Furthermore, it contains high levels of phytonutrients, such as fatty acids, phenolics, and organic acids.^6,23^ RO is valued for its possible therapeutic benefits and anti-inflammatory and antimicrobial properties.^25^

RO has been employed to treat wounds and/or scars for many years due to its high concentration of essential fatty and unsaturated acids necessary for cell membrane permeability and wound healing processes.^29^ In a previous study, the herbal ointment containing rosehip extract showed significantly increased wound healing in second–degree burns compared to silver sulfadiazine.^1^

RO has demonstrated significant antiulcerogenic properties. The ability of *Rosa canina* to completely stop ulcer formation in rat models was initially noted by Gürbüz in 2003.^9^ According to Lattanzio et al, *Rosa canina* administration prevented the creation of haemorrhagic ulcers and the degradation of the stomach mucosa.^14^

Moreover, the antibacterial and antifungal activity of clotrimazole-rosehip oil nanoethosomes hydrogel was investigated. The results showed that the hydrogel was beneficial in treating oral thrush and gingivitis and that individuals were more compliant with its administration.^33^

A recent systematic review shows insufficient data supports RO in healing wounds. More research is necessary to prove this action and its possible application as a component in topical preparations.^2^

To date, fewer studies have been conducted on the effects of ROs in dentistry, and its ability to enhance the healing of oral ulcers remains a topic of ongoing research. Thus, the study aimed to investigate the anti-ulcerative impact of rosehip oil emulsion on induced oral ulcers and its efficiency in healing.

## MATERIALS AND METHODS

### Preparation for ROE

Pure rosehip oil was used to formulate rosehip oil emulsion (ROE) at the Department of Chemistry, Faculty of Science, Al Azhar University.^21^

### Proliferation of Human Gingival Fibroblast (HGF)

ROE at a concentration of 20 µg/mL was incubated with HGF cells *in vitro* and co-cultivated for 24, 48, and 72 h to determine the effect of the produced ROE on cell proliferation. The cells were finally stained using the Vybrant® MTT Cell Proliferation assay to detect the proliferation effect.

### Induction of Ulcer and Grouping

The Research Ethics Committee approved the study, Faculty of Dental Medicine for Girls, Al Azhar University (REC code: P-PD-24-04). Using the prior research findings, G*Power version 3.1.9.7 calculated the sample size, which was estimated to be 66 rats.^18^

From Theodor Bilharzias (Giza, Egypt), 66 adult male albino rats weighing between 200 and 250 g were obtained. The rats were anaesthetised, and 0.12% chlorhexidine digluconate was used to sterilise the mucosa. The chemically induced ulceration was made on the left cheek mucosa by placing a 6 × 6 mm round filter paper immersed in 50% acetic acid for 30 to 60 s. The operation technique was standardised for all the animals.^27^

#### Animal grouping

Animals were randomly divided into a control group of 33 rats that developed ulcer formation without treatment. The test group consists of 33 rats that received topical treatment of 20 mg/ml of ROE after ulcer formation, 3 times/day for 10 days.^42^ Then, animals in both groups were sacrificed, and the specimens were collected on days 3, 7, and 10 to undergo histological and histomorphometric examination.

#### Histological examination

After being promptly preserved in 10% neutral buffered formalin, the mucosal tissues were divided into sections that were 5 µm thick. The sections were stained using H&E according to standard protocol, Masson’s trichrome, and underwent histomorphometric examination.

#### Histomorphometric examination

Five non-overlapping microscopic fields for each slide were photomicrographed by a digital camera mounted on a research light microscope (Leica Qwin 500, England). Then, the percentage area (%) of new collagen formation within the specimen was automatically calculated.

### Statistical Analysis

Data were summarised as mean and SD values, and then one-way ANOVA was applied to compare groups with a significance level at P ≤ 0.05, followed by Tukey’s post hoc test for pairwise comparison.

## RESULTS

### Results of HGF Proliferation

**Fig 1:**
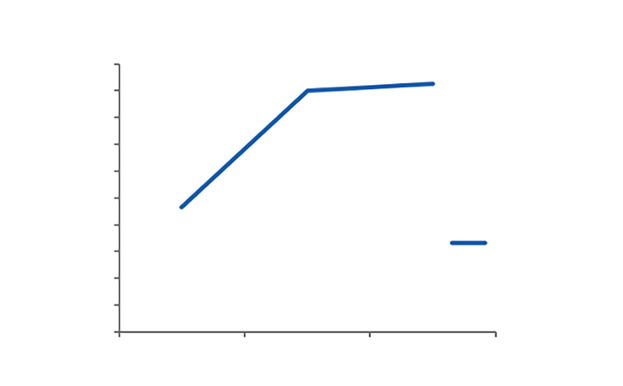
Mean and SD of cell proliferation of fibroblast cells at a concentration of 20 µg/mL rosehip oil emulsion at different intervals.

MTT detection findings showed that ROE could stimulate cell proliferation. The optical density of cells at 570 nm was 0.93 ± 0.19 at 24 h, then increased to 1.80 ± 0.16 at 48 h, and finally reached its maximum increase of 1.85 ± 0.28 at 72 h. The mean optic density (OD) at 24 h differed statistically significantly from the mean OD at the other time intervals, but there was no significant difference at 48 or 72 h (Fig 1).

### Hematoxylin and Eosin (H&E)

**Fig 2a and b:**
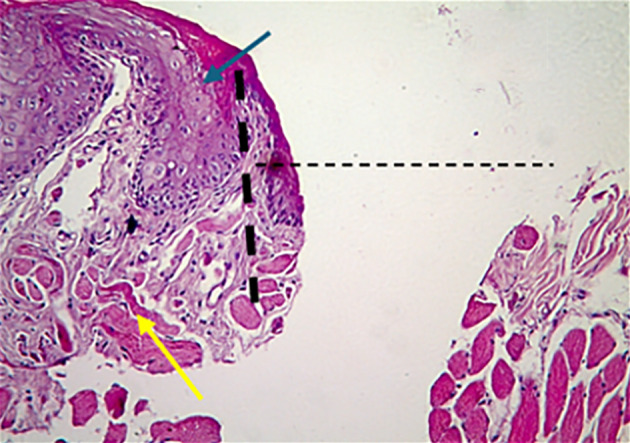

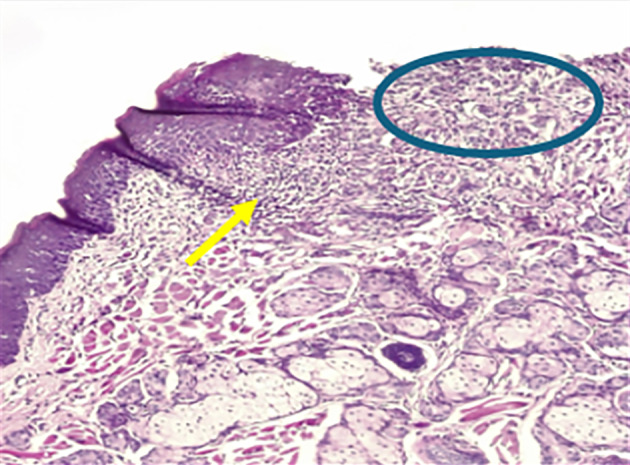
A photomicrograph of the buccal mucosa of rat. (a) The control group on day 3 showed a large epithelium defect (dashed line), the epithelium at the margin enrolled into the defect (blue arrow), and CT revealed a spread of inflammatory cells in between the muscles (yellow arrow). (b) The treated group on day 3 showed a defect containing granulation tissue (circle); the defect was smaller than the control group, and CT showed more inflammatory cells (yellow arrow) (H&E, ×100)

**Fig 3a and b:**
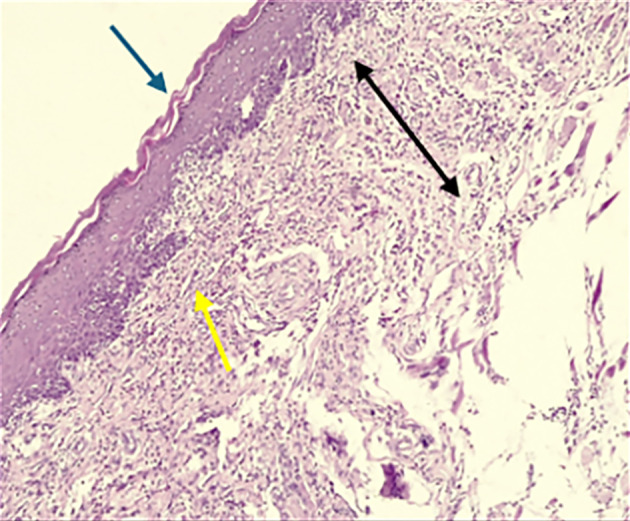

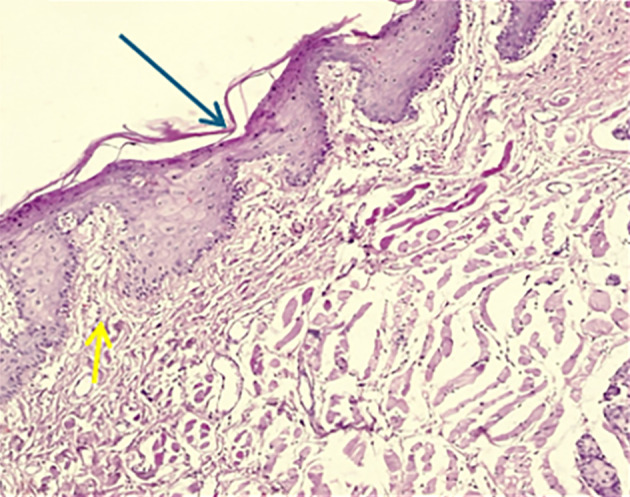
A photomicrograph of the buccal mucosa of a rat. (a) The control group on day 7 showed intact epithelium with irregular basement membrane (blue arrow), CT filled with dense inflammatory cell infiltrate (yellow arrow), and increased thickness of CT with loss of architecture of underlying muscle (black arrow). (b) The treated group on day 7 showed intact epithelium and underlying CT (blue arrow); the epithelium showed increased thickness with apparent rete pegs and scarce inflammatory cells (yellow arrow) (H&E, ×100)

**Fig 4a and b:**
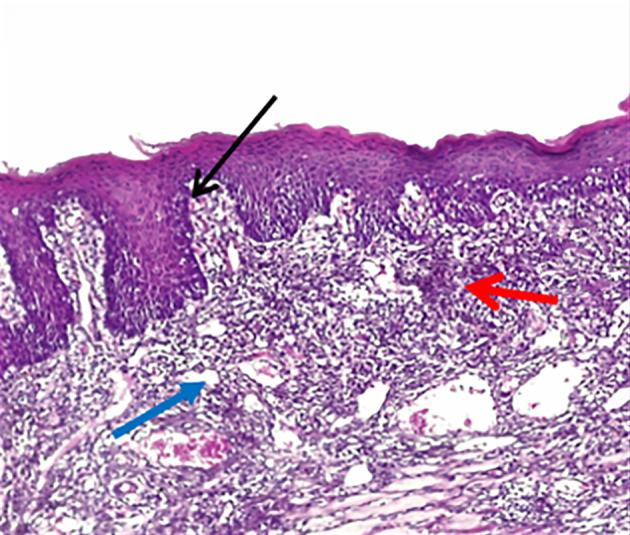

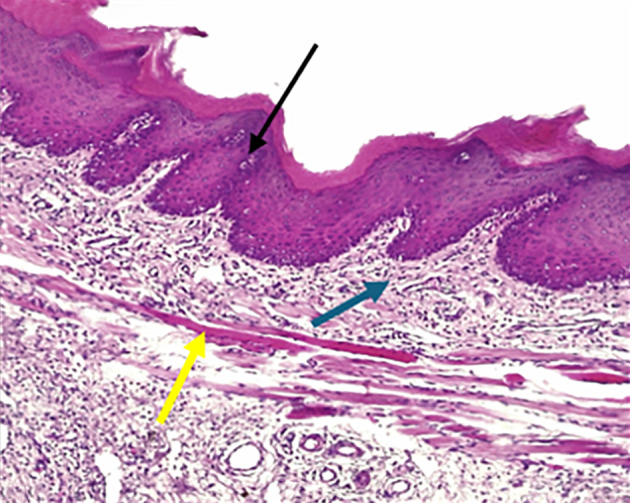
A photomicrograph of the buccal mucosa of rat. (a) The control group at day 10 showed closed epithelium covered by a relatively thick layer of keratin, with apparent rete pegs (black arrow), and CT showed heavy inflammatory cells (red arrow) and contained interstitial tissue space ( blue arrow). (b) The treated group showed closed epithelium covered with a thicker keratin layer (black arrow), the muscle in the underlying CT runs horizontally (yellow arrow), and the CT showed well-organised collagen fibres (blue arrow). (H&E, ×100)

According to the results of HE stains, the control group had a more profound defect on D3. In contrast, in the treated group, the defect was filled with granulation tissue with more inflammatory cells in the connective tissue (Fig 2). On D7, there was thin epithelium with an irregular basement membrane and more inflammatory cells in connective tissue in the control group, while the treated group had moderately thick keratinised stratified epithelium with long rete pegs (Fig 3). On D10, the ulcers of the two groups were healed, and the treated group showed the thickest keratin and epithelium layer with the muscle running horizontally in the underlying CT, well-organised collagen fibres, and fewer inflammatory cells than the control group (Fig 4).

### Masson’s Trichrome Special Stain

**Fig 5a to f:**
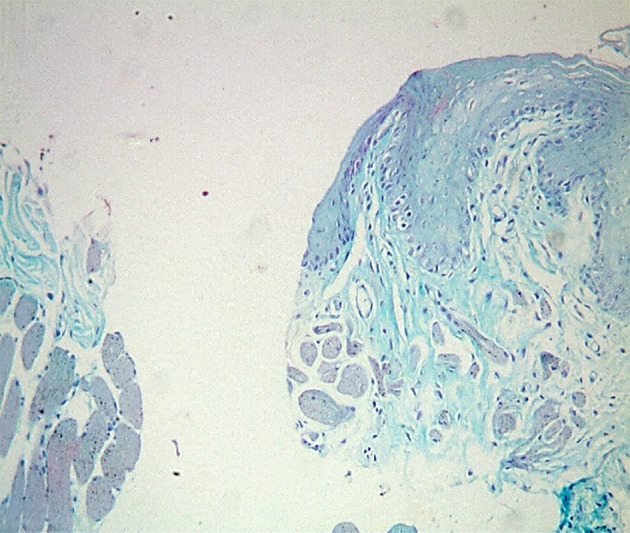

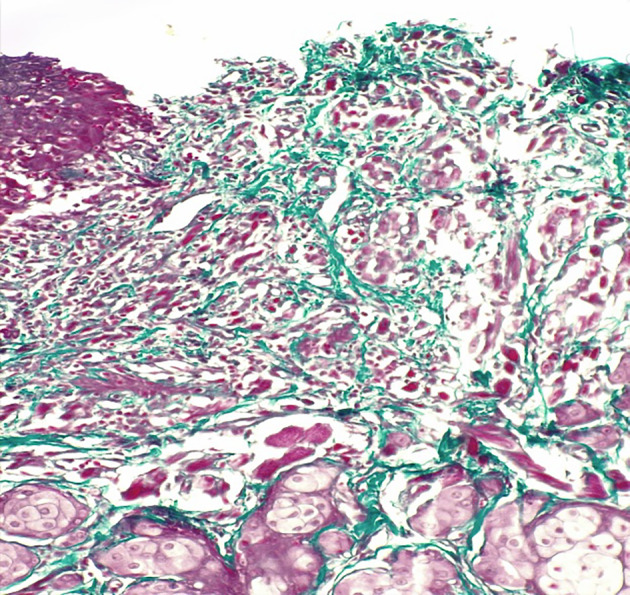

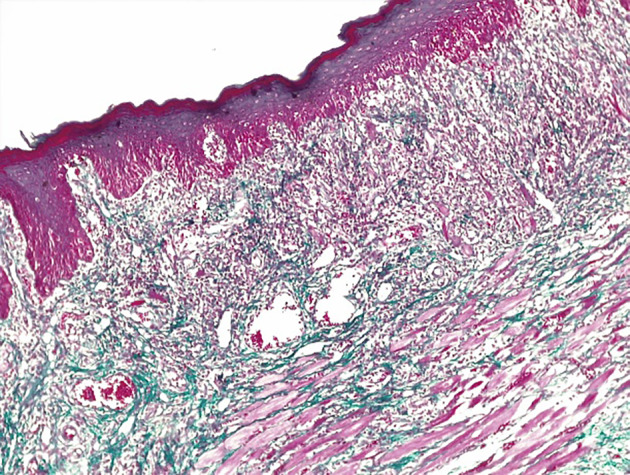

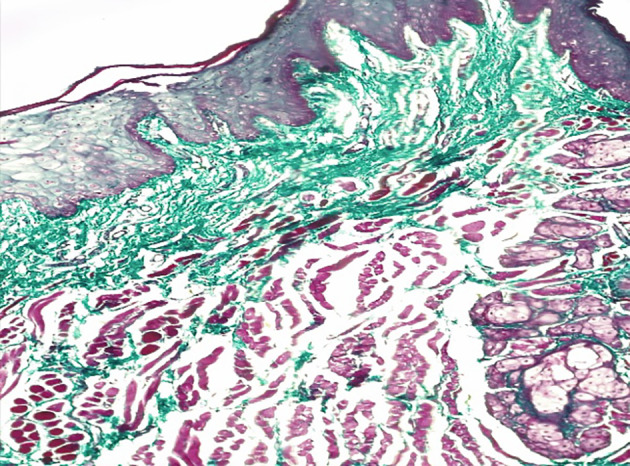

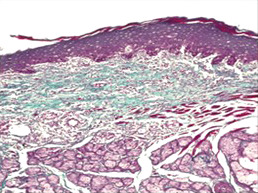

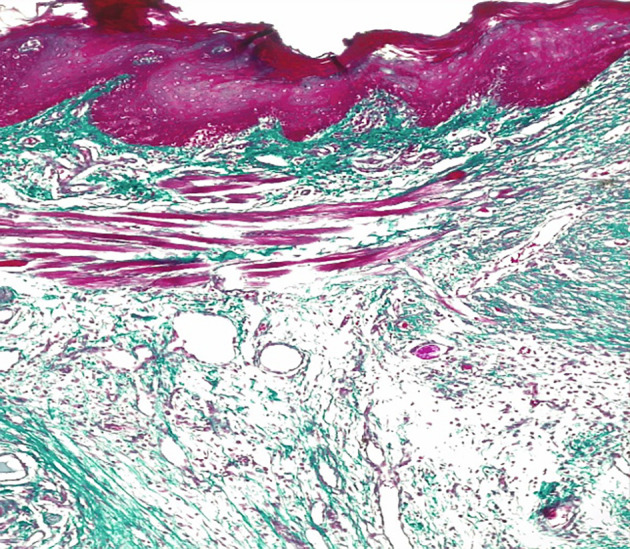
A photomicrograph of rat buccal mucosa. (a) Control group at day 3 showing a large epithelium defect with collagen fibres at the periphery. (b) The treated group on day 3 showed a defect almost filled with granulation tissue, mainly collagen. (c) The control group on day 7 showed widespread, thin, randomly arranged collagen fibres in the CT. (d) The treated group on day 7 showed coarse, radially organised collagen bundles stained green. (e) The control group at day 10 showed horizontally arranged dense and thick collagen bundles. (f) The treated group at day 10 showed widespread, densely packed, coarse, and organised collagen bundles (Masson trichrome, ×100).

On day 3 (D3), the control group showed a large defect with collagen fibres at the periphery of the epithelial defect. In comparison, the treated group showed the defect almost entirely contained within the epithelium. On day 7(D7), the collagen fibres were thinly, randomly arranged in the CT in the control group compared with the coarse, radially organised bundles in the treated group. On day 10 (D10), the control group showed horizontally arranged dense and thick collagen bundles, while the treated group had a wide spread of densely packed, coarse, and well-organised collagen bundles (Fig 5).

### Histomorphometeric Results

**Fig 6:**
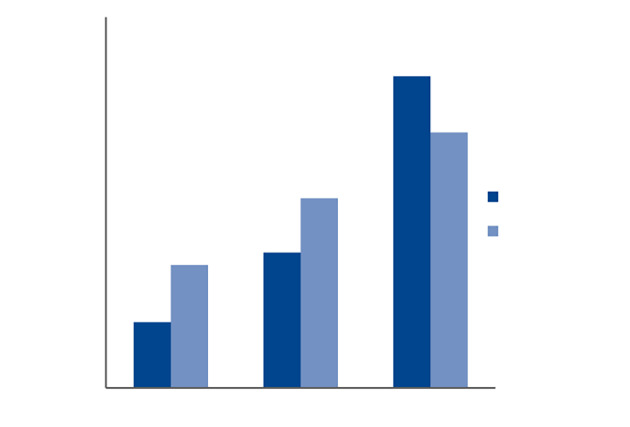
Percentage of collagen formation (%) in Masson staining of two groups at different time intervals.

By estimating the area % of collagen formation, we observed that at D3 and D7, the mean area of collagen formation in the treated group was statistically significantly more than that of the control group, with P values of 0.02 and 0.006, respectively. While at D10, the difference between the two groups was not statistically significant (Table 1 and Fig 6).

**Table 1:** The means and SD for % of collagen formation of the two groups

	Day 3	Day 7	Day 10
Control group	7.1± 3.9	14.6±1.23	33.7±5.38
Treated group	13.3±2.36	20.5±2.58	27.6±3.65
P value	0.02*	0.006*	0.1
*Significance at P <0.05. Tukey’s post hoc test for pairwise comparison.

## DISCUSSION

The development of more efficient therapeutic approaches to managing oral ulcers is dictated by and needs considerable interest in healing research. Rosehip oil emulsion was reported here for the first time to estimate the anti-ulcerative impact of this herbal emulsion on the healing process of oral ulcers. The rat model was selected for this study because of its suitable size, simple manipulation, accessibility in therapy, and compatibility with human anatomy, physiology, and genetics.^11,31,39^

This study required an oral ulceration model to test the effectiveness of herbal emulsions, especially chemical-induced ones, because this was the most popular way of induction in rats according to a previous systematic review.^27^

Recent research has shown that rosehip extracts are highly suitable options for topical usage in the form of nanoemulsions.^24^ In the present study, chitosan-loaded RHE was prepared to increase oil wettability and decrease surface tension. Chitosan has superior biocompatibility and biodegradability features, and it firmly adheres to the negatively charged mucus membrane, thus improving cell absorption of oil.^28^

The results of MTT detection revealed RHE can significantly increase the proliferation of HGF at 24 h (0.93 ± 0.19), then to 1.80 ± 0.16 at 48 h, and the maximum increase was achieved (1.85 ± 0.28) at 72 h, which provides essential data for the subsequent animal experiments.

Regarding H&E staining, the treated group on day 3 showed the defect containing granulation tissue and more inflammatory cells compared to the control group because, during the initial phase of the ulcer, an inflammatory response with gathered inflammatory cells aiding in the phagocytic process promotes the removal of injured tissue and the start of wound repair.^12^

On the other hand, on days 7 and 10, the treated group showed increased epithelium thickness with apparent rete pegs, indicating mucosal epithelial healing with suppressed inflammatory cells compared to the control group. The degree of inflammation has been observed to significantly decrease in the middle and late phases of ulcer healing.^32^

The positive effect observed in the treated group may be attributed to the regulation of the inflammatory response, as it has been demonstrated that this RHE exhibits anti-inflammatory activity by inhibiting inflammatory mediators such as IL-1R and IL-1.^4,15^ Moreover, rosehip oil may promote the shift of macrophages from M1 (CCR7+/CD68+) to M2 (CD163+/CD68+).^16^

Our observations are consistent with a previous study that revealed that topical treatment with *Rosa rubiginosa* oil enhanced skin wound healing in both diabetic and control rats, as indicated by a decrease in the levels of inflammatory mediators like TNFα, IL-1β, and IL-6 and an improvement in wound healing.^22^ Previous *in-vitro* and *in-vivo* clinical trials have shown rosehip seed oil’s ability to inhibit chemotaxis.^5,13^

Interestingly, both groups have similar formation of new blood vessels, as RHO slightly altered the expression of the vascular endothelial growth factor (VEGF) proteins in mouse skin.^38^ Moreover, the highest vitamin C content is found in rosehips (*Rosa canina*), which have 1252.3 mg/100 g fresh weight.^28^ Vitamin C boosts collagen synthesis in tissues and eliminates free radicals as an antioxidant, leading to additional benefits of wound healing.^30^

Several of its components have been identified as antioxidant and antimicrobial, including fatty acids, polyphenols, vitamins B, C, and E, and carotenoids.^16,19^ These RO ingredients enable a more effective healing process, mainly when utilised early. The healing process depends on the biosynthesis and deposition of collagen, as well as its subsequent maturation. Our results showed that at D3 and D7, collagen formation of the treated group was statistically significantly higher than that of the control group at P values (0.02, 0.006), respectively. While at D10, the difference between the two groups was not statistically significant.

This may contribute to RO enhancing the synthesis of collagen, especially type III, and prevent TGF-β1 and α-SMA from being expressed,^16^ promoting collagen formation in the early to intermediate phases of ulcer healing. RO was found to elevate the skin’s insulin-like growth factor-1 (IGF-1) level, further contributing to hair growth.^36^ IGF-1 is a profibrogenic growth factor that stimulates the proliferation of fibroblasts and keratinocytes, extracellular matrix deposition, and collagen synthesis.^37,41^

On day 10, the amount of collagen in the treated group decreased compared to the control group, but didn’t reach a significant level. It may indicate that the treated group is in the late stages of ulcer healing. The remodelling or maturation that occurs during the final phase of healing is marked by a controlled reduction in the quantity of collagen and a modification in the type and manner of arrangement of collagen.^3^

## CONCLUSIONS

The topical application of 20 mg/mL ROE possesses anti-inflammatory properties, increasing the thickness of epithelium, promoting production, and remodelling the collagen. Consequently, enhancing healing processes. This suggests that it may be effective as a topical treatment for managing oral ulcers.

### Acknowledgements

This work was performed at Faculty of Dental Medicine for Girls, Al Azhar University, Cairo, Egypt.
